# Upregulation of extracellular proteins in a mouse model of Alzheimer’s disease

**DOI:** 10.1038/s41598-023-33677-z

**Published:** 2023-04-28

**Authors:** Sangkyu Kim, Jessica Fuselier, Anna Latoff, Justin Manges, S. Michal Jazwinski, Andrea Zsombok

**Affiliations:** 1 Tulane Center for Aging and Deming Department of Medicine, Tulane University Health Sciences Center, New Orleans, LA USA; 2 Tulane Center for Aging and Department of Physiology, Tulane University Health Sciences Center, New Orleans, LA USA; 3 Deming Department of Medicine, Tulane University Health Sciences Center, 1430 Tulane Ave., MBC 8513, New Orleans, LA 70112 USA; 4 Present Address: Data Science Department, Catalytic Data Science, Charleston, SC USA

**Keywords:** Genetics, Neuroscience, Systems biology, Biomarkers, Diseases, Neurology, Risk factors

## Abstract

Various risk factors of Alzheimer’s disease (AD) are known, such as advanced age, possession of certain genetic variants, accumulation of toxic amyloid-β (Aβ) peptides, and unhealthy lifestyle. An estimate of heritability of AD ranges from 0.13 to 0.25, indicating that its phenotypic variation is accounted for mostly by non-genetic factors. DNA methylation is regarded as an epigenetic mechanism that interfaces the genome with non-genetic factors. The Tg2576 mouse model has been insightful in AD research. These transgenic mice express a mutant form of human amyloid precursor protein linked to familial AD. At 9–13 months of age, these mice show elevated levels of Aβ peptides and cognitive impairment. The current literature lacks integrative multiomics of the animal model. We applied transcriptomics and DNA methylomics to the same brain samples from ~ 11-month-old transgenic mice. We found that genes involved in extracellular matrix structures and functions are transcriptionally upregulated, and genes involved in extracellular protein secretion and localization are differentially methylated in the transgenic mice. Integrative analysis found enrichment of GO terms related to memory and synaptic functionability. Our results indicate a possibility of transcriptional modulation by DNA methylation underlying AD neuropathology.

## Introduction

Dementia is the chronic loss of normal cognition and disruption of cognitive functions accompanied by personality changes. Disrupting most cognitive activities, it affects autonomous daily life. AD is the most common form of dementia prevalent among older people. It is associated with age-related changes in the nervous system, including accumulation of extracellular Aβ peptides and plaques and intracellular neurofibrillary tau tangles^1–3^. Risk factors include possession of certain genetic variants, head injuries, cardiovascular disorders, and unhealthy lifestyles^4,5^. In addition, infection by certain pathogenic microbes may contribute to AD development^6,7^. Therapies targeting Aβ, tau, and other modifiable risk factors are being pursued, but effective cures are yet to be established^8,9^.

Toxic Aβ peptides, such as Aβ_1–40_ (Aβ40) and Aβ_1–42_ (Aβ42), have been taking center stage in AD research. These are cleavage products of amyloid precursor protein (APP), a single-pass transmembrane protein of unknown function found in many tissues^10^. Many gene mutations have been reported that increase the production of the toxic peptides^11^. Soluble Aβ monomers and oligomers are aggregated to insoluble fibrils or plaques^12^. Controversy exists over the form of detrimental Aβ and its cellular location. Regardless, a plethora of data suggests a causal role of toxic Aβ in AD pathology. For example, transgenic mice carrying mutant versions of the human *APP* gene linked to familial AD develop pathological phenotypes reminiscent of AD^13,14^. These phenotypes include increased Aβ levels, age-related neurocognitive deficits, and reduced viability.

Phenotypic variation in AD is largely accounted for by non-genetic factors. Genes in co-expression networks of human frontal cortex samples show complex relationships with pathological and clinical traits^15^. These genes are hierarchically clustered into many modules where no individual genes dominate in gene-trait relationships. Even the *e4* allele of *APOE*, the best-known genetic risk factor, accounts for only 2–5% of phenotypic variances. Multiomics of human AD brains, which shows various subnetworks of SNPs, transcripts, and proteins, includes a small subnetwork containing *APOE*^16^. According to a large-scale-data compilation, the heritability of AD ranges from 0.13 to 0.25^17^. Thus, most of the AD cases belong to the non-familial, sporadic type.

As AD is a complex disease affected by many external factors impinging on related but heterogeneous mechanisms, integrative multiomics is likely to greatly enhance the accuracy and interpretability of data. The Tg2576 mice produce a mutant version of human *APP* containing the Swedish double mutation (KM670/671NL)^18^. At 9–13 months of age, these mice show cognitive impairment and increased levels of Aβ peptides and plaques in the brain. Transcriptomics on this mouse model has been reported^19,20^, but literature is scarce on DNA methylomics or integrative multiomics applied to the same brain samples.

Using the Tg2576 model, we investigated differential gene expression, coupled with differential DNA methylation. Our results indicate that the transgenic mice are enriched in transcripts involved in extracellular matrix structures and functions. Similar results were obtained with DNA methylomics. Furthermore, a subset of transcripts that is predictive of a related DNA methylation profile was enriched in GO terms related to memory and synaptic function. These results suggest a possibility of transcriptional modulation of genes involved in neuropathology of AD by DNA methylation.

## Methods

### Animals

Male and female wild type (Wt) and Tg2576 (Tg) mice were ordered from Taconic (Stock #1349). Animals were housed in 12-h dark–light cycle rooms at room temperature and had free access to water and standard rodent chow. Under deep anesthesia with isoflurance, the mice (11–14 month old) were decapitated with a rodent guillotine, the brain was removed and micro-dissected. Dissections of brain regions containing both dorsal hippocampus and cortex were frozen and kept at – 80 °C until further processing. Thus, each brain biospecimen used in this study is a mixture of both brain regions that were processed together for DNA and RNA extraction. All procedures were performed in accordance with the NIH Guide for the Care and Use of Laboratory Animals and approved by the Institutional Animal Care and Use Committee of Tulane University. This multiomics study, reported in accordance with ARRIVE guidelines, analyzed a total of 13 mice, five Wt (three female) and eight Tg (four female) (Supplementary Table S1).

### RNA-seq

For RNA-seq, we analyzed data from 12 samples (5 Wt and 7 Tg mice) that passed preprocessing (Supplementary Table S1). Total RNA was extracted from the brain section containing hippocampus and cortex using RiboPure™ RNA Purification Kit (ThermoFisher Scientific). Ribosomal RNA was removed using Ambion RiboMinus Eukaryote System. RNA concentration and integrity were checked using an Agilent Bioanalyzer (RIN > 7). Libraries were prepared using Ion Total RNA-Seq Kit for the AB Library Builder System, and transcript sequence reads were generated using the Ion Torrent S5XL sequencing platform. The average number of total reads was 33.2 million per sample. Sequence reads were aligned and mapped to mm10 FASTA and GTF files using STAR and Bowtie 2 embedded in the Ion Torrent RNASeqAnalysis plugin. Using edgeR, transcript count data were subjected to gene annotation, removal of low count genes, normalization to remove biases across libraries^21^. The edgeR-treated data set was analyzed for differential expression between Wt and Tg groups using DESeq2^22^, with adjustment for sex and batch (Supplementary Methods).

Differentially expressed genes (DEGs) were analyzed for associated gene ontology (GO) terms using R packages gprofiler2, clusterProfiler, GeneTonic, and enrichGO^23–25^. In addition, the output of DESeq2 analysis was subjected to gene set enrichment analysis (GSEA) using the R fgsea package^26^. GSEA requires genes ranked according to a standardized statistical score. To rank genes, we used the Wald statistic (= ‘stat’), which is log_2_[fold change] (lfc) divided by its standard error. Curated mouse gene sets in canonical pathways were downloaded from the GSEA Molecular Signature Database (MSigDB) (https://www.gsea-msigdb.org/gsea/msigdb/mouse_geneset_resources.jsp). Currently, the mouse MSigDB offers gene sets from Reactome, BioCarta, and WikiPathways databases. In the fgsea package, enrichment score (ES) is calculated in the same way as in Broad Institute GSEA^27^. Normalized enrichment scores (NES) were generated by dividing ES by the mean values of permutated ES (n = 1000).

### Weighted gene co-expression network analysis (WGCNA)

The edgeR-processed data from the Tg and Wt mice were analyzed using the R package WGCNA^28^. The soft thresholding power was set empirically at 22, and either signed or unsigned network adjacency matrix and topological overlap matrix (TOM) were calculated. A signed network treats only positively correlated nodes as connected whereas an unsigned network treats both positively and negatively correlated nodes as connected. Co-expressed genes were hierarchically clustered using the dissimilarity structure from the signed or unsigned TOM and the “average” agglomeration method. Modules of dissimilarity < 0.1 were merged. Module eigengene, gene significance, and module membership were calculated for each module using functions provided by WGCNA. A module eigengene refers to the 1st principal component of a module, gene significance is the correlation between expression profiles of genes in a module and the trait, and the module membership is the correlation between the module eigengene and the gene expression profile. GO analysis was applied to modules of interest, as described above for transcriptomics.

### DNA methylomics

For DNA methylomics, we analyzed data from 12 samples (4 Wt and 8 Tg mice) that passed sequence preprocessing, 11 of which were also analyzed for RNA-seq (Supplementary Table S1). Mouse genomic DNA (≥ 500 ng), prepared using Quick-DNA Microprep Plus Kit, Zymo Research, was sent to Zymo Research for Classic RRBS (Reduced representation bisulfite sequencing). The bisulfite sequencing service included bisulfite treatment, library construction and quality control, paired-end data generation (Illumina HiSeq 2000), data alignment and mapping (using Bismark), and methylation calling (using Bismark Methylation Extractor). The total number of reads was 21 million per sample on average and the average mapping efficiency to the mm10 reference genome was 68%. Various types of data were delivered, including trimmed FASTQ and BAM files.

The BAM files were analyzed using the R package methylKit^29^. Differentially methylated CpG sites between Tg and Wt groups were identified with adjustment for sex. A differentially methylated site (DMS) was regarded as a CpG dinucleotide with (1) the difference in mean DNA methylation levels greater than 10% between Tg and Wt groups and (2) FDR lower than 0.1. Significant CpG sites were linked to the nearest transcription start sites using org.Mm.eg.db, and genomation^30^. GO terms associated with the linked genes were retrieved using the ClusterProfiler package. Functional relations of genes (“Gene-Concept Networks”) within or between significant GO categories were inferred and visualized using the R enrichplot package.

### Statistical analysis

Transcript and DNA methylation data were also analyzed using partial least squares (PLS) methods. PLS-discriminant analysis (PLS-DA) and sparse PLS were applied to transcript data using R package mixOmics^31^. PLS was run for the binary classification through a dummy matrix that was treated as a continuous variable^32^. Sparse PLS through LASSO penalization was applied for feature selection.

Integrative analysis was applied to data from 11 brain biospecimens (4 Wt and 7 Tg mice) from which both transcriptomics and DNA methylomics data were obtained (Supplementary Table S1). The two-way orthogonal PLS method (O_2_PLS) models the variation of two different datasets in three parts: a joint part, an orthogonal (systemic) part, and a random part. The joint part accounts for variation in both datasets, the orthogonal part is unique to each dataset, and the random part corresponds to residual variation. Datasets in the joint part are highly correlated. The R OmicsPLS package was used to model the joint variation between the transcript and the DNA methylation datasets^33^. Transcripts and DNA methylation sites whose levels highly covary were selected using DIABLO (Data Integration Analysis for Biomarker discovery using a Latent cOmponents) provided by the MixOmics package^34^. DIABLO is a multiomics integrative method based on PLS. We analyzed the first two principal PLS components for feature selection and GO analysis. Gene ontology terms representing the selected features were retrieved as described above.

## Results

### Transcriptomics

Using DESeq2, we found expression of 100 genes statistically significant between Tg and Wt mice (Supplemental Table S2). Of these, expression of 84 genes was up regulated (lfc ranging from 0.35 to 18.51, adjusted p-value (padj) < 0.01), and the other 16 were down regulated (lfc ranging from − 18.62 to − 0.46, padj < 0.01) in the transgenic group.

**Figure 1:**
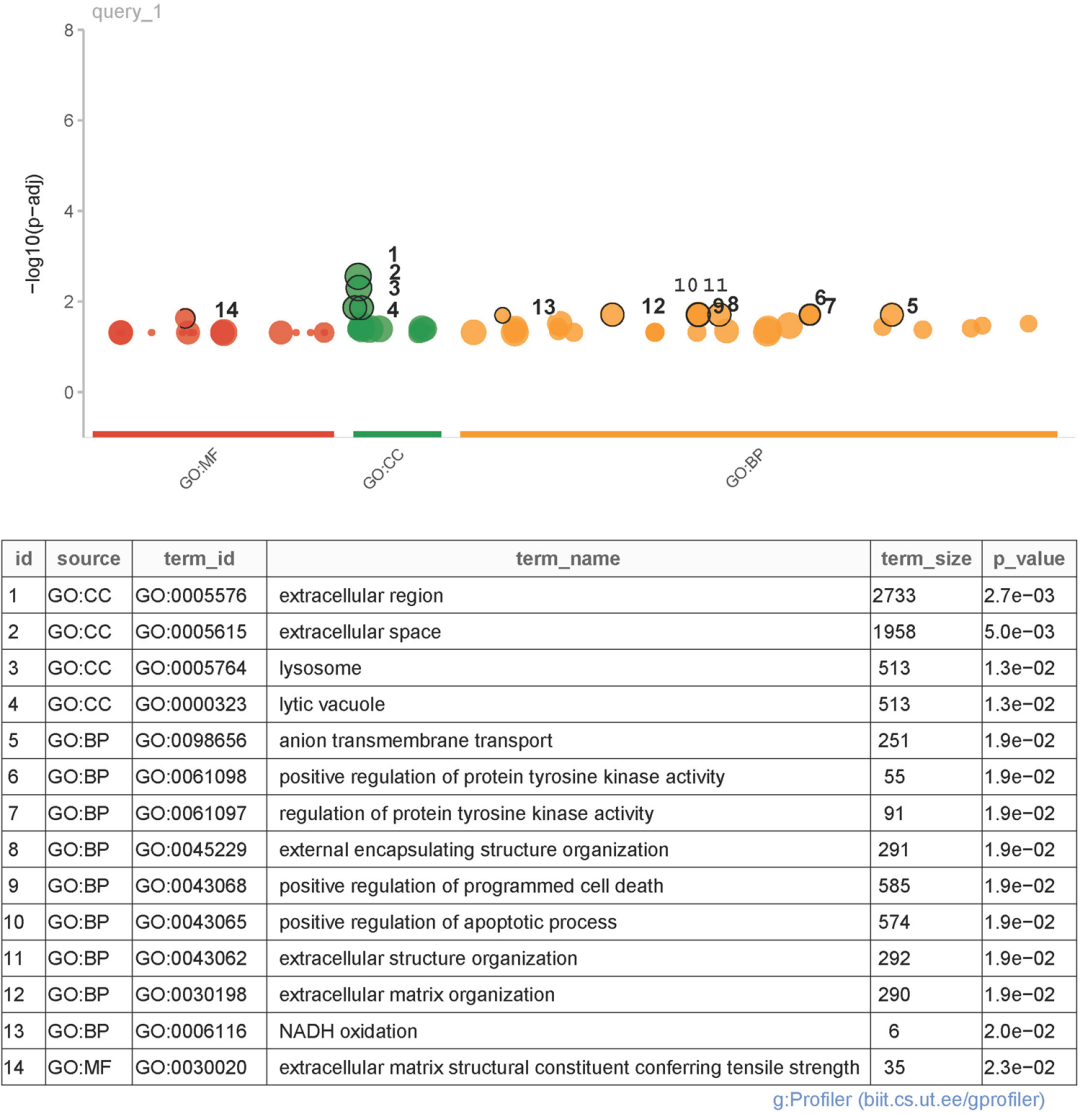
Significant GO terms output by gprofiler2 (only top 14 terms are shown here; see Supplementary Table S3 for the full list). The upper plot is a graphical visualization of the bottom table. The numbers in the plot correspond to the id’s in the table; source = GO categories (*BP* biological process, *CC* cellular component, *MF* molecular function), *term_size* number of genes that are annotated to the term, *p_value* FDR-adjusted pval.

The DESeq2-identified DEGs were analyzed for associated GO terms. Of the 65 GO terms with padj < 0.05 (Supplemental Table S3), the two most significant ones were cellular components (CC) ‘extracellular region’ and ‘extracellular space’ (Fig. 1). Biological processes (BP) or a molecular function (MF) related to the extracellular terms were also found down the list.

**Figure 2:**
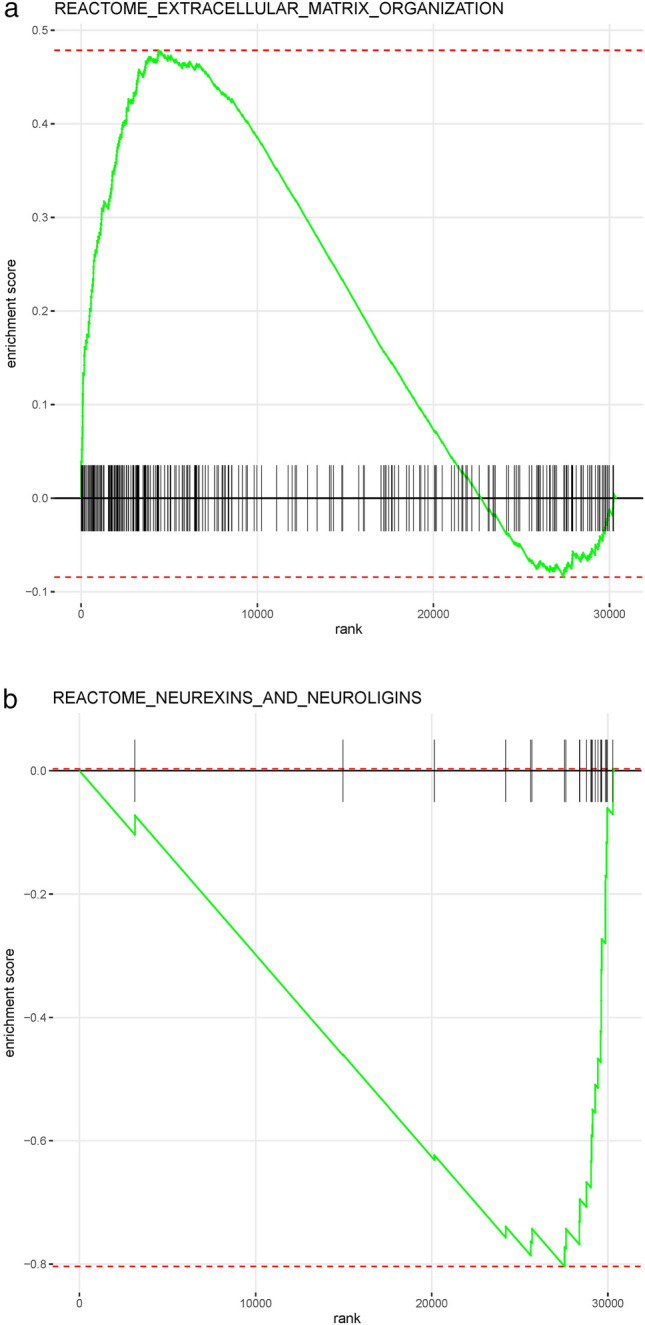
Changes in ES value (y axis) as GSEA was run down the ordered gene list (x axis). The score at the highest or lowest peak of the plot is the ES score for the gene set. (**a**) ES = 0.479 (Table 1) for the Reactome pathway Extracellular matrix organization (R-HAS-1474244); (**b**) ES = − 0.804 for Neurexins and neuroligins (R-HAS-6794361).

In addition to the gene ontology analysis, functional gene set enrichment analysis was applied to the DESeq2 output using curated mouse gene sets from the Reactome pathway database. Of the 69 pathways with padj < 0.01, the top eight pathways with NES greater than 2 were all closely related to structures or functions of extracellular components or domains (Table 1). On the other hand, most of the 14 pathways with NES lower than -2 were related to neurotransmission and neuronal signaling. These results indicate that in Tg mice, genes involved in extracellular matrix structures or functions are up regulated (Fig. 2a) while those involved in neuronal signaling are down regulated (Fig. 2b).

**Table 1:** Reactome pathways with NES > 2 or < − 2.

Reactome pathway	pval	padj	ES	NES
Collagen degradation	5.68E−08	5.40E−06	0.70	2.35
Integrin cell surface interactions	1.66E−07	1.26E−05	0.59	2.20
Laminin interactions	2.97E−06	1.08E−04	0.74	2.18
Assembly of collagen fibrils and other multimeric structures	8.04E−07	4.34E−05	0.61	2.16
Extracellular matrix organization	9.14E−12	2.32E−09	0.48	2.11
Cholesterol biosynthesis	4.41E−05	8.81E−04	0.70	2.05
Collagen chain trimerization	5.79E−05	1.07E−03	0.61	2.04
NCAM1 interactions	9.33E−05	1.56E−03	0.72	2.03
Collagen biosynthesis and modifying enzymes	1.83E−05	4.64E−04	0.56	2.02
Collagen formation	5.44E−06	1.65E−04	0.53	2.00
RAF activation	1.69E−04	2.44E−03	− 0.60	− 2.04
Transmission across chemical synapses	8.58E−10	1.63E−07	− 0.46	− 2.09
HATS acetylate histones	7.63E−05	1.36E−03	− 0.64	− 2.10
MAPK targets nuclear events mediated by map kinases	1.73E−04	2.44E−03	− 0.66	− 2.12
Synaptic adhesion like molecules	2.16E−04	2.88E−03	− 0.69	− 2.12
Signaling by NTRKS	1.61E−06	6.81E−05	− 0.54	− 2.13
CRMPS in sema3a signaling	9.47E−05	1.56E−03	− 0.76	− 2.14
Trafficking of AMPA receptors	7.73E−05	1.36E−03	− 0.76	− 2.16
Signaling by NTRK1 TRKA	2.83E−06	1.08E−04	− 0.57	− 2.17
Neurotransmitter receptors and postsynaptic signal transmission	7.89E−09	8.57E−07	− 0.50	− 2.17
Unblocking of NMDA receptors glutamate binding and activation	3.44E−05	7.26E−04	-0.72	− 2.24
Neuronal system	5.47E−16	4.16E−13	− 0.47	− 2.26
Activation of NMDA receptors and postsynaptic events	1.14E−06	5.09E−05	− 0.71	− 2.33
Protein protein interactions at synapses	5.87E−09	7.43E−07	− 0.63	− 2.41
Neurexins and neuroligins	2.84E−09	4.31E−07	− 0.80	− 2.59

*pval* enrichment p-value, *padj* Benjamini-Hochberg-adjusted p-value, *ES* enrichment score, *NES* normalized ES (ES divided by the mean ES from 1000 random samples).

**Figure 3:**
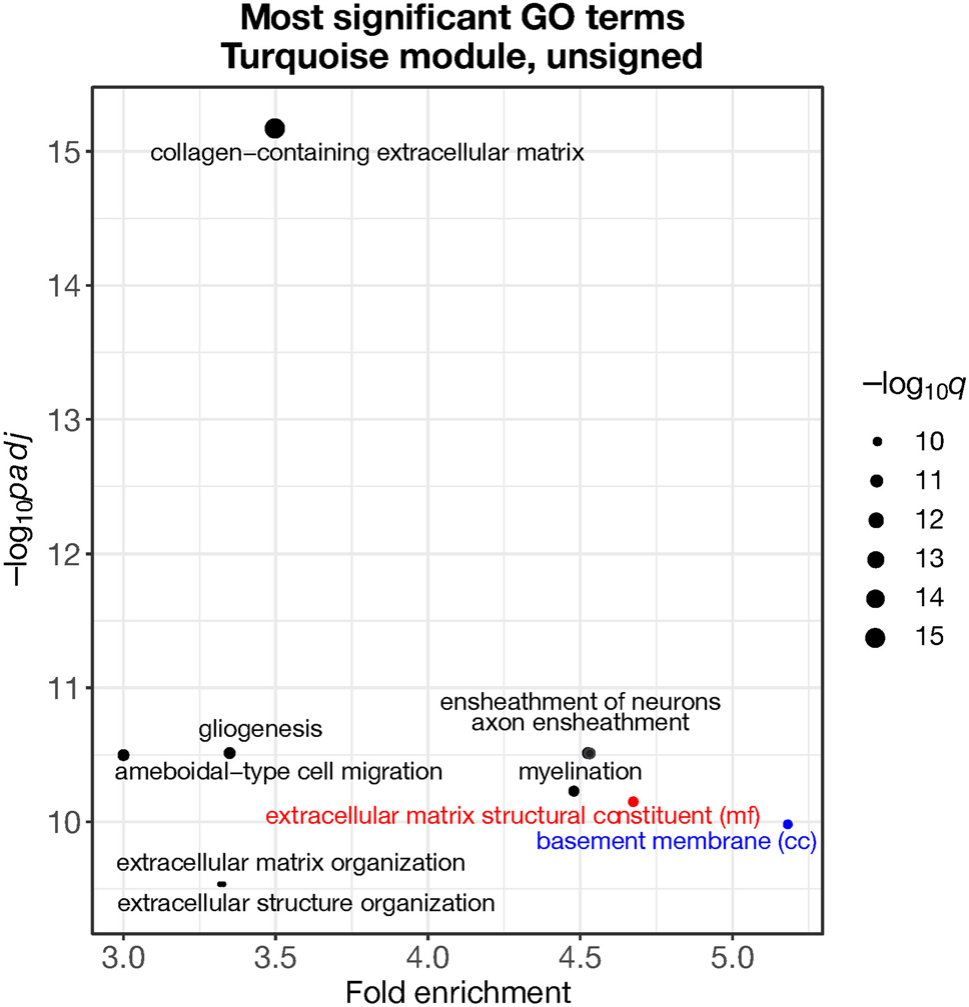
A bubble plot of top 10 GO terms enriched among the genes in the turquoise module. Fold enrichment is the ratio of the proportion of input genes annotated in each GO term to the proportion of all genes annotated in the same term; padj = Benjamini–Hochberg-adjusted pval; q = FDR-adjusted pval; and all terms belong to biological process except the ones marked cc (cellular component) and mf (molecular function).

GSEA may allow for characterization of genes in well-defined biological processes or pathways, in which participating genes are likely to be coordinately expressed. Using WGCNA, it is possible to first group genes by their expression profiles and then characterize clusters of genes with similar expression profiles. Modules of interconnected genes were generated by clustering genes based on a signed or unsigned network using the R package WGCNA^28^. Of the unsigned-network modules, we chose a module labeled ‘turquoise’ for further analysis, which showed the highest module-trait relationship (Spearman’s ρ = − 0.56, pval = 0.057; Supplemental Fig. S1). The turquoise module, consisting of 1377 genes, showed a significant correlation between the intramodular connectivity and gene significance (Pearson’s r = 0.4, p = 4.7e−54; Supplemental Fig. S2). Genes in this module were associated with GO terms related to extracellular organization and neural cell differentiation (Fig. 3).

Among the signed-network modules, ‘yellow’ and ‘slategray’ were the top two with the highest module-trait relationship (ρ = − 0.58, pval = 0.05 and ρ = − 0.56, pval = 0.058, respectively; Supplemental Fig. S3). In both modules, module membership and gene significance were significantly correlated (yellow, r = 0.33, p = 3.6e−30; slategray, ρ = 0.49, p = 5.5e−08). The yellow module, consisting of 1132 genes, was associated with GO terms similar to those from the unsigned network module (Supplemental Fig. S4). No GO terms were found associated with the slategray module.

### DNA methylomics

We found 443 DMSs (Diff_10per.csv in Supplementary Data), which were linked to 182 annotated genes (dms_443-gene_182.csv in Supplementary Data). Of these, 71 genes were linked to hypermethylated DMSs and 111 genes were linked to hypomethylated DMSs. GO terms significantly associated with the mapped genes (padj < 0.01) include those related to protein secretion and extracellular localization (Table 2; Supplemental Fig. S5).

**Table 2:** GO terms enriched in 182 genes annotated with 443 DMSs.

ID	Description (all BP)	pval	padj
GO:0009306	Protein secretion	8.11E−07	0.001
GO:0035592	Establishment of protein localization to extracellular region	8.34E−07	0.001
GO:0071692	Protein localization to extracellular region	1.01E−06	0.001
GO:0050708	Regulation of protein secretion	5.57E−06	0.0032
GO:0051703	Intraspecies interaction between organisms	5.63E−06	0.0032
GO:0009914	Hormone transport	6.84E−06	0.0032
GO:0030073	Insulin secretion	1.22E−05	0.0049
GO:0030072	Peptide hormone secretion	1.42E−05	0.0051
GO:0002791	Regulation of peptide secretion	1.73E−05	0.0055
GO:0046879	Hormone secretion	2.76E−05	0.0079
GO:1902904	Negative regulation of supramolecular fiber organization	4.04E−05	0.0098
GO:0032309	Icosanoid secretion	4.14E−05	0.0098
GO:0032303	Regulation of icosanoid secretion	4.49E−05	0.0098

*DMS* differentially methylated site, *BP* Biological process, *pval* enrichment p-value, *padj* Benjamini–Hochberg-adjusted p-value. The whole output is in ego_10p.csv in Supplementary Data.

### Integrative PLS

Integrative analysis was applied to data from 11 brain biospecimen from which both transcriptomics and DNA methylomics data were obtained. Unsupervised decomposition of the variations in the transcript and DNA methylation data sets generated a single joint partition, which accounts for about 50% of the variation in the transcript data and 14% of the variation in the methylation data. The O_2_PLS model also yielded an orthogonal partition that is specific to 12% of the transcript data only; there was no orthogonal partition specific to the DNA methylation data. Thus, unlike the transcript data set, most of the variation (~ 86%) in DNA methylation data was estimated to be stochastic; the remaining small portion was predicted to co-vary with transcript data.

**Figure 4:**
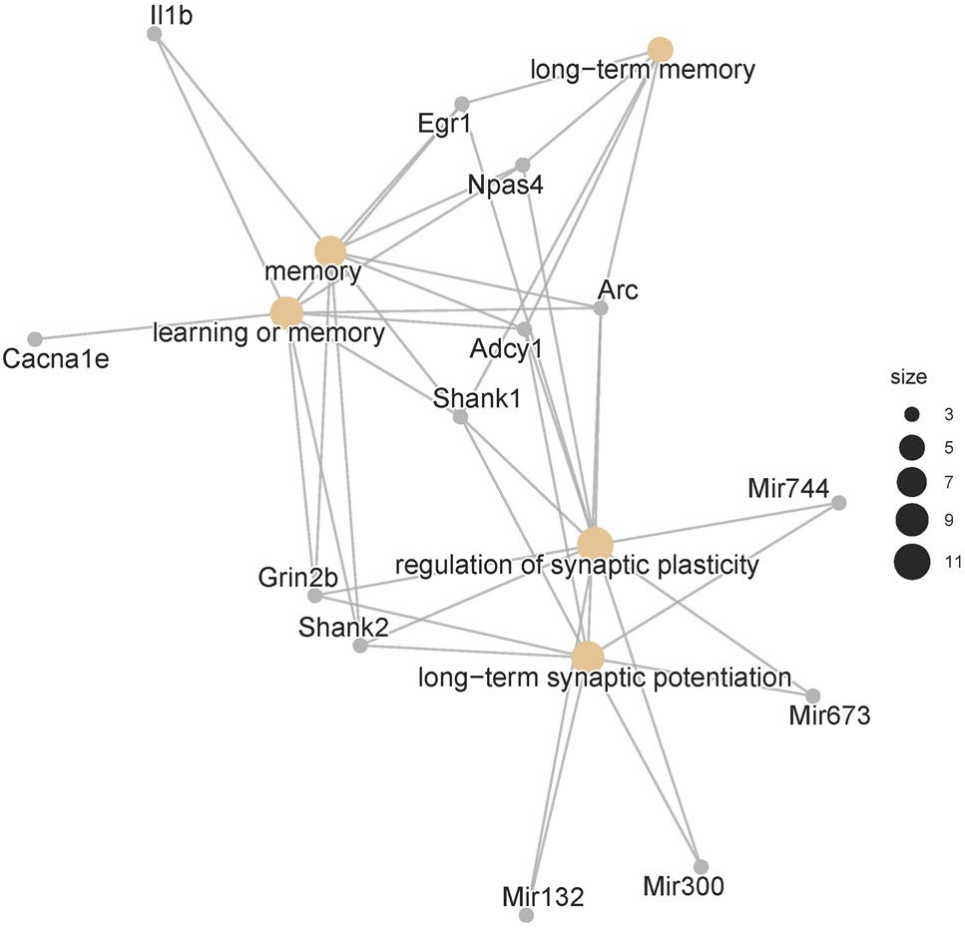
A “cnetplot” displaying the top five GO terms and related genes in the input gene list (ego_diablo_r2.csv in Supplementary Data). It was generated using the cnetplot function available in the R enrichplot package. “size” refers to the number of genes assigned to each GO category.

Key features common to both data sets were identified using a supervised method that integrates multiomics data sets. The goal was to characterize the first two pairs of latent components that discriminate the binary trait. We selected 500 top-loading transcripts from the 1st component and the same number of transcripts from the 2nd components, which were mapped to 395 and 376 gene subsets, respectively. No GO terms were found associated with the 1st subset, but the 2nd subset was associated with GO terms containing memory, synaptic plasticity, and potentiation (Fig. 4). The same number of DNA methylation sites were selected from the 1^st^ and 2^nd^ components, which were linked to 123 and 127 nearest gene subsets. No GO terms were significantly associated with the two subsets.

## Discussion

Multiomics heavily depends on analytical tools. Therefore, we devoted a substantial portion of our discussion to some important aspects of our analytical approaches that may not be immediately obvious. We also presented literature-based speculations and possibilities, which we hope will lead to testable hypotheses.

### Single omics: extracellular localization/organization vs. synaptic function

We found 100 DEGs from DESeq2 analysis of brain sample data obtained from ~ 1-year-old Tg and Wt mice. These DEGs were characterized by GO terms related to extracellular localization and organization. Similar enrichment terms were found with genes in co-expression modules that showed a significant correlation between the intramodular connectivity and gene significance.

The GO results were further supported by the results of functional GSEA based on the Reactome database. Most of the Reactome pathways with top positive enrichment scores were related to interactions or organization of extracellular components. These results indicate that in Tg mice, biological pathways involved in extracellular structures and functions are upregulated. Interestingly, Reactome profiles at the negative end of the ranked gene list were distinctively different: Most of the pathways with top negative enrichment scores were related to neurotransmission and synaptic signaling. For GSEA, we ranked genes in the decreasing order by “stat,” which is standardized log_2_(fold change). Positive stat values reflect increased expression whereas negative stat values reflect decreased expression of the corresponding genes in Tg mice. Thus, pathways listed at the positive end of the ordered gene list represent upregulated ones whereas those listed at the negative end represent downregulated ones. In other words, we speculate that in Tg mice, the Reactome pathways related to extracellular structures or functions might have been upregulated whereas those related to neurotransmission or neuronal signaling were downregulated. This implieates that upregulation of the extracellular matrix pathways might underlie downregulation of the signaling function in the Tg mice expressing the human *APP* gene carrying the Swedish mutation. Upregulation of extracellular matrix pathways or components has been observed in human AD brain transcriptomics, cell cultures expressing a mutant version of PSEN1^35,36^, and in AD brain proteomics^37^. Downregulation of genes involved in synaptic functions coupled with upregulation of ECM genes was also observed, which concurs with our GSEA results^35^.

Of the 182 genes (dms_443-gene_182.csv in Supplementary Data) identified by DNA methylation data analysis, 71 genes were linked to hypermethylated DMSs and 111 genes were linked to hypomethylated DMSs. Hypermethylation is generally regarded to be associated with transcriptional downregulation, and vice versa. However, studies going against the generalization exist, in which hypermethylation is associated with upregulated gene expression^38,39^. As the number of this type of DNA methylation studies increases, we expect to encounter more similar unconventional cases. There is another reason for considering all the mapped genes together regardless of their association directions with gene expression data. Components in a biological pathway can be correlated with one another positively or negatively (e.g., A activates B, but B represses C, etc.). This is why the ‘unsigned’ co-expression network described for WGCNA includes all the genes whose expression levels are correlated either way. Furthermore, most of the mapped genes were linked to single DMSs, and it is hard to imagine that a single-site methylation change would significantly affect expression of a nearby gene (unless it is experimentally proven to have such an influential role).

The GO terms that were significantly associated with these genes include those related to extracellular localization of proteins, which is concomitant with those obtained from transcriptomics. Thus, results of individual omics pose the possibility that transcription of genes, especially those located in extracellular matrix, might be modulated by differential DNA methylation.

### Integrative multiomics: the possibility of transcriptional modulation by DNA methylation

An unsupervised method predicted a joint partition that could account for 50% of the variation in the transcript data and 14% of the variation in the DNA methylation data. The two types of data in the joint partition are predictive of each other. When the ‘inter-associations’ between the two datasets were directed to be discriminant, we have somewhat complicated results. From the analysis of the 1st component pair, we found that DMS-linked genes or transcript-mapped genes were not enriched in any GO terms. Likewise, the DMS-linked genes from the 2nd component pair were not enriched in any GO terms. Only the transcript-mapped genes from the 2nd component pair were enriched in GO terms related to memory and synaptic function, which is in line with the pathophysiology of AD.

In evaluating the results of the integrative analyses, we consider three things. First, the statistical failure to detect significant GO terms of interest in a set of genes doesn’t necessarily mean the total absence of related biological functions. This is an especially reasonable consideration in a study with limited power. Second, the multivariate-based integration method we used (DIABLO) is an extension of sparse generalized canonical correlation analysis (sGCCA), which maximizes covariance between linear combinations (latent components) of variables^34^. In other words, the method is based on multivariate correlation. Correlation of two different types of data sets may have little to do with their functional relatedness because the data sets may randomly correlate with each other without any functional or mechanistic relatedness. This is highly probable with high-dimensional data structures. If so, collected gene subsets will be unrelated to each other, and GO analysis will produce different results, if any. Third, correct identification of DMS-target genes is impossible without experimental evidence. Identification of differentially expressed genes through transcriptomics is straightforward because transcripts are direct products of corresponding genes. On the other hand, bioinformatic identification of a gene that is functionally linked to one or more DMSs is not straightforward because it currently depends on their location relative to nearby genes. If a DMS lies within a gene body (38% of the 443 DMSs), such as in an exon or intron, it is probably correct to functionally link the DMS to the gene where it lies. However, if a DMS lies outside of any gene body (39%), mapping of such a DMS is not as straightforward. We followed the default delineation of a promoter (± 1000 bp around the transcription start sites). All the other DMSs (23%) were regarded as intergenic. Thus, even if we have a set of genes that are differentially methylated as well as differentially expressed, distance-based DMS-linking is unlikely to identify all the genes correctly, which will affect subsequent gene enrichment analysis. Therefore, given the results of the supervised integrative analysis, we cannot rule out the possibility that the two omics data sets may contain one or more functionally or mechanistically related data blocks.

We examined overlapping genes between differential expression and DNA methylation by taking two approaches. First, we made a summary table (Supplementary Table S4) that lists significant DEGs (differentially expressed genes identified by RNA-seq) and genes linked to significant DMSs (differentially methylated sites identified by BS-seq). This table shows 84 transcriptionally upregulated and 16 downregulated genes (from Supplementary Table S2). The table also shows 182 annotated genes (71 hypermethylated and 111 hypomethylated) (dms_443-gene_182.cvs in Supplementary Data), from 443 DMSs identified as significant using methylKit (Diff_10per.csv in Supplementary Data, which show all the DNA methylation and genome mapping information). None of the genes are common in any cross comparisons. Second, we made another summary table (Supplementary Table S5) that lists commonly occurring genes in comparisons of RNA component 1—DNA methylation component 1 and RNA component 2—DNA methylation component 2 identified by DIABLO. From each RNA component, 500 features were selected, and their annotated genes were identified (228 genes for RNA component 1 in diablo_r1.csv in Supplementary Data; 233 genes for RNA component 2 in diablo_r2.csv for RNA component 2). Likewise, gene names linked to DMSs were identified (diablo_d1_gene.csv for component 1 and diablo_d2_gene.csv for component 2). There is one gene (histocompatibility 13) common between RNA component 1 and DNA methylation component 1, and five (chromogranin B, Zmynd12, Fa2, Pigg, and Fam124a) between RNA component 2 and DNA methylation component 2. Of these, chromogranin B is predicted to be in the extracellular matrix, which can be regarded as supporting the possibility of transcriptional modulation by DNA methylation. However, results obtained from many related genes are more reliable than those obtained from shortlists of genes, not to mention the time and effort taken for manual search and curation.

### AD pathology by soluble Aβ

Two molecular hallmarks of AD pathology are extracellular amyloid plaques and intracellular neurofibrillary tau tangles^40^. The amyloid cascade hypothesis assumes that Aβ peptide fragments, such Aβ40 and Aβ42 produced by the amyloidogenic cleavage of APP, aggregate in neurotoxic amyloid plaques, leading to the neuropathology of AD^41^. Thus, mainstream research on AD has been to elucidate the pathogenic effects of Aβ peptides and plaques on brain functions^1–3^. Several lines of evidence indicate that it is soluble oligomeric forms of Aβ that are responsible for most of the pathologies of AD. The Aβ42 level correlates with the disease severity^42^. It has more potent effects on neuronal functions and cell viability^43–45^. The ratio of Aβ42 to Aβ40 under pathological conditions change in favor of greater Aβ42^46^. Soluble Aβ promotes hyperphosphorylation of tau protein, promoting the formation of neurofibrillary tangles and neurite atrophy^47^. On the other hand, the level of insoluble forms is not a reliable correlate with disease severity^48^. Moreover, Aβ plaques are observed postmortem in clinically normal individuals^49^.

Similar findings have been reported with Tg2576 mice. In these mice, the level of soluble Aβ begins to increase from 6 to 8 months of age, and insoluble amyloid plaques are noticeable beginning at 9–12 months^50–52^. The appearance of insoluble plaques seemed to coincide with the onset of memory loss, but no correlation was observed in a sample of old and young mice^53^. These observations led to the hypothesis that Aβ intermediates between monomers and insoluble aggregates are responsible for neuropathology in Tg mice^53^.

### Genetic and non-genetic contributors to toxic Aβ generation and accumulation

Our study explored the possibility of DNA methylation as an epigenetic mechanism, based on the Aβ hypothesis of the disease, using the mouse model. According to the modified Aβ hypothesis in which soluble Aβ is the culprit, biological or clinical manifestation of AD pathologies depends on the rate of toxic soluble Aβ production from APP and its level in the central nervous system. The production rate will depend on time (age), but time is neither a reactant nor a catalyst of the cleavage reaction. Both genetic and environmental risk factors may affect the production rate of soluble Aβ by increasing the substrate (APP). This hypothesis assumes that the amyloidogenic pathway is in operation to some degree even under normal conditions. For example, individuals with Down syndrome (DS) are likely to produce excess APP because Down syndrome is genetically characterized by an extra copy of chromosome 21 where *APP* is located. Indeed, brain extracts from individuals with DS (58–80 years of age at death) showed elevated levels of soluble Aβ^54^. Increased ratios of Aβ42 to Aβ40 were observed in blood samples taken from live DS patients with dementia (20–52 years age range) compared with DS patients without dementia (13–51 years age range)^55^.

The production rate of toxic Aβ can be increased by genetic mutations that lead to preferential activation of the amyloidogenic pathway. For example, certain mutations in *APP*, *PSEN1*, or *PSEN2* are known to increase the amount of toxic Aβ*.* The Swedish mutation increases the initial cleavage of APP at the β-cleavage site over the non-amyloidogenic α-cleavage site. *PSEN1* and *PSEN2* encode presenilin 1 and 2, which are subunits of the gamma secretase complex, and there are mutations in these genes that result in increased production of toxic Aβ. Human astrocytes expressing APOE4 showed lower clearance of soluble Aβ than those expressing the other alleles of APOE^56^. Thus, in principle, the presence of one or more potent risk factors, like the genetic mutations seen in familial AD, may set disease onset relatively early in life.

On the other hand, the effects of environmental and lifestyle risk factors are usually so small, or often negligible, that the disease would progress gradually and subtly over several decades. Most AD cases (more than 90%) belong to this type of sporadic AD, occurring late in life seemingly in an age-dependent way^57^. While familial AD is mostly due to mutations in *APP*, *PSEN1*, and *PSEN2* genes, sporadic AD is determined by many genetic and non-genetic factors over a long period of time. Note that accumulation of toxic Aβ may underlie sporadic AD too^58^. Our study suggests DNA methylation as an epigenetic mechanism relaying non-genetic effects to the genome.

### Toxic Aβ and extracellular matrix

Expression of the human *APP* gene with the Swedish mutation (*APPsw*) in Tg mice is associated with significant changes in expression of other genes. Bioinformatic compilation of the GO terms and Reactome pathways related to ECM and neuronal signaling is based on relevant publications in the literature. Soluble Aβ peptides are secreted into the extracellular space^12^. Composed mostly of various glycoprotein components, ECM fills the extracellular space, around neurons, glia, and synaptic junctions. ECM components interact with neural cell surface receptors and affect almost all aspects of nervous system function, including cell migration and adhesion, axonal guidance, and formation of synapses and neural circuits^59,60^. Furthermore, expression of various ECM components increases as the disease progresses, leading to neuronal atrophy and synaptic dysfunction^61^. These findings largely concur with our GSEA results.

### Toxic Aβ and DNA methylation

One plausible mechanism that may mediate the effects of Aβ on gene expression is DNA methylation. In *APPsw*-expressing human glioblastoma (H4-sw) cells, expression of *IGFBP3* was reduced and its promoter was hypermethylated, compared to normal cells (H4)^62^. Reduced *IGFBP3* expression and the promoter hypermethylation were also observed with Aβ42-treated H4 cells. On the other hand, expression of *HMOX1* was increased, and DMSs in its promoter were hypomethylated in H4-sw cells and Aβ42-treated H4 cells^63^. In both genes, gene expression and DNA methylation levels were reversed by treatment of cells with a DNA methylation inhibitor. These results suggest transcriptional modulation of genes by differential DNA methylation. These results also suggest locally varying effects of Aβ oligomers on DNA methylation. Aβ oligomers can induce global hypomethylation in cultured hippocampal cells by reducing DNMT activity (based on an in-vitro DNMT activity assay applied to nuclear extracts)^64^. Application of the methyl donor folic acid to the cells restored the methylation status and DNMT activity in a dose-dependent manner. Loss of function mutations in *TET* genes, which encode dioxygenases involved in DNA demethylation, also leads to global hypomethylation with local hypermethylation^65^.

### ROS may mediate DNA methylation by Aβ

How could extracellular Aβ mediate its effect on genomic DNA methylation? We speculate that reactive oxygen species (ROS) could be a potential mediator. Reactive oxygen species (ROS) are signaling molecules. Human Aβ can directly generate superoxide from metal ions (deposited extracellularly in senile plaques) through the Fenton reaction^66,67^. Extracellular superoxide undergoes rapid dismutation to H_2_O_2_, which can diffuse freely through cell membranes as a relatively stable ROS. However, a biologically controllable source of cellular ROS is the cell membrane-bound NADPH oxidases (NOX). NOX activities are inducible by extracellular Aβ^68^. Soluble Aβ induces oxidative stress and vascular dysfunction in the brain, the severity of which is significantly diminished by chemical or genetic inhibition of NOX in rats and mice^69–71^. In human and rodent brain tissues, ROS generated by Aβ-activated NOX can lead to endothelin release, which triggers pericyte-mediated capillary constriction^72^.

Evidence also exists for the role of ROS in differential DNA methylation. ROS can induce epigenetic changes, often in response to exposure to external or environmental stimuli, such as ionizing radiation, exposure to airborne nanoparticles, herbicides, or heavy metals^73,74^.

## Conclusions

Our results suggest that in Tg mice expressing the human mutant APP, biological pathways impacting ECM structures and functions are upregulated whereas those in neurotransmission or neuronal signaling are downregulated. Both single omics and integrative multiomics suggest the possibility of transcriptional modulation by DNA methylation. We regard ROS a mediator between toxic Aβ and differential DNA methylation.

### Study limitations

There are limitations that need to be addressed. First, due to the limited sample size, our study is potentially liable to variation in the results and biological interpretation. Second, the brain samples we used in this study are mixtures of hippocampus and cortex. Transcriptome and epigenome profiles vary temporally and spatially. Thus, variation in the source and collection time of specimens across different studies limits comparisons and interpretations of the results. Third, details of the neuropathology underlying the model may differ from those of human AD. For example, Tg2576 mice do not develop neurofibrillary tau tangles in the brain^75^. Fourth, the study was limited to one timepoint. Despite these limitations, our study raises the possibility of transcriptional modulation by DNA methylation in a model of Alzheimer’s disease.

## Supplementary Information

Supplementary Information 1.

Supplementary Information 2.

## Data Availability

The datasets generated and analyzed during this study are available in the Gene Expression Omnibus (GEO) repository with Accession Numbers GSE223417 for RNA-seq and GSE223349 for BS-seq data.
