# Author Correction: Upregulation of extracellular proteins in a mouse model of Alzheimer’s disease

**DOI:** 10.1038/s41598-023-35102-x

**Published:** 2023-05-17

**Authors:** Sangkyu Kim, Jessica Fuselier, Anna Latoff, Justin Manges, S. Michal Jazwinski, Andrea Zsombok

**Affiliations:** 1 Tulane Center for Aging and Deming Department of Medicine, Tulane University Health Sciences Center, New Orleans, LA USA; 2 Tulane Center for Aging and Department of Physiology, Tulane University Health Sciences Center, New Orleans, LA USA; 3 Deming Department of Medicine, Tulane University Health Sciences Center, 1430 Tulane Ave., MBC 8513, New Orleans, LA 70112 USA; 4 Present Address: Data Science Department, Catalytic Data Science, Charleston, SC USA

Correction to: *Scientific Reports* 10.1038/s41598-023-33677-z, published online 28 April 2023

The original version of this Article contained an error in the Data availability section.

“The datasets generated and analyzed during this study are available in the Gene Expression Omnibus (GEO) repository with Accession Numbers GSE223417 for RNA-seq and GSE223348 for BS-seq data.”

now reads:

“The datasets generated and analyzed during this study are available in the Gene Expression Omnibus (GEO) repository with Accession Numbers GSE223417 for RNA-seq and GSE223349 for BS-seq data.”

The original Article has been corrected.

