# Supplementary material for: Upregulation of extracellular proteins in a mouse model of Alzheimer’s disease
Source: Sci Rep. 2023 Apr 28;13:6998. doi: 10.1038/s41598-023-33677-z (PMC10147640; doi:10.1038/s41598-023-33677-z)
Supplement: Supplementary file 1 — Supplementary Information 1. [file 41598_2023_33677_MOESM1_ESM.docx]

**Contents of Supplementary Information**

# A. Supplementary tables and figures

# B. Supplementary Methods

# C. Supplementary Data (in a separate folder)

# A. Supplementary tables and figures

**Supplementary Table S1** Mouse samples used in this study

| sample ID | group | sex | RNA-Seq | Bisulfite-seq | DIABLO |
| --- | --- | --- | --- | --- | --- |
| 12 | Wt | female | yes | yes | yes |
| 32 | Tg | female | yes | yes | yes |
| 41 | Wt | male | yes | yes | yes |
| 48 | Tg | male | no | yes | no |
| 67 | Tg | male | yes | yes | yes |
| 68 | Tg | male | yes | yes | yes |
| 69 | Tg | male | yes | yes | yes |
| 79 | Wt | female | yes | yes | yes |
| 80 | Wt | female | yes | yes | yes |
| 91 | Tg | female | yes | yes | yes |
| 92 | Tg | female | yes | yes | yes |
| 93 | Tg | female | yes | yes | yes |
| 95 | Wt | male | yes | no | no |

All mice are ~ 11-months- old; Bisulfite-seq = BS-seq for DNA methylomics; DIABLO is an integrative method used for combined analysis of both omics data. Refer to the main text for details).

**Supplementary Table S2** 100 DEGs output by DESeq2

| Gene | lfc | lfcSE | stat | pval | padj |
| --- | --- | --- | --- | --- | --- |
| App | 1.65 | 0.142 | 11.68 | 1.55E-31 | 4.69E-27 |
| Gm8215 | 18.51 | 3.13 | 5.91 | 3.34E-09 | 8.87E-06 |
| Gm24515 | 18.39 | 3.177 | 5.79 | 7.16E-09 | 8.87E-06 |
| Agr2 | 18.39 | 3.177 | 5.79 | 7.17E-09 | 8.87E-06 |
| Gm10375 | 18.39 | 3.177 | 5.79 | 7.17E-09 | 8.87E-06 |
| Gm11313 | 18.39 | 3.177 | 5.79 | 7.17E-09 | 8.87E-06 |
| Gm15169 | 18.39 | 3.177 | 5.79 | 7.17E-09 | 8.87E-06 |
| Gm17171 | 18.39 | 3.177 | 5.79 | 7.17E-09 | 8.87E-06 |
| Gm24135 | 18.39 | 3.177 | 5.79 | 7.17E-09 | 8.87E-06 |
| Gm24368 | 18.39 | 3.177 | 5.79 | 7.17E-09 | 8.87E-06 |
| Olfr314 | 18.39 | 3.177 | 5.79 | 7.17E-09 | 8.87E-06 |
| Tdpoz4 | 18.39 | 3.177 | 5.79 | 7.17E-09 | 8.87E-06 |
| Prlr | 1.78 | 0.302 | 5.91 | 3.45E-09 | 8.87E-06 |
| Prnp | 0.82 | 0.138 | 5.93 | 3.00E-09 | 8.87E-06 |
| 2010110K18Rik | 17.86 | 3.178 | 5.62 | 1.91E-08 | 1.49E-05 |
| 2210010C04Rik | 17.86 | 3.178 | 5.62 | 1.91E-08 | 1.49E-05 |
| Bpifa6 | 17.86 | 3.178 | 5.62 | 1.91E-08 | 1.49E-05 |
| D930032P07Rik | 17.86 | 3.178 | 5.62 | 1.91E-08 | 1.49E-05 |
| Gm11771 | 17.86 | 3.178 | 5.62 | 1.91E-08 | 1.49E-05 |
| Gm15658 | 17.86 | 3.178 | 5.62 | 1.91E-08 | 1.49E-05 |
| Gm23275 | 17.86 | 3.178 | 5.62 | 1.91E-08 | 1.49E-05 |
| Gm26147 | 17.86 | 3.178 | 5.62 | 1.91E-08 | 1.49E-05 |
| H2-M10.6 | 17.86 | 3.178 | 5.62 | 1.91E-08 | 1.49E-05 |
| RP23-402K6.2 | 17.86 | 3.178 | 5.62 | 1.91E-08 | 1.49E-05 |
| RP24-329K22.1 | 17.86 | 3.178 | 5.62 | 1.91E-08 | 1.49E-05 |
| Ssxb2 | 17.86 | 3.178 | 5.62 | 1.91E-08 | 1.49E-05 |
| Tff3 | 17.86 | 3.178 | 5.62 | 1.91E-08 | 1.49E-05 |
| Xlr3e-ps | 17.86 | 3.178 | 5.62 | 1.91E-08 | 1.49E-05 |
| Slc37a2 | 1.13 | 0.211 | 5.37 | 7.92E-08 | 6.02E-05 |
| Gm26232 | 0.79 | 0.156 | 5.08 | 3.87E-07 | 2.87E-04 |
| Gfap | 1.00 | 0.203 | 4.91 | 8.92E-07 | 6.45E-04 |
| Gm24305 | 0.52 | 0.106 | 4.87 | 1.14E-06 | 8.07E-04 |
| Gm25813 | 0.87 | 0.185 | 4.72 | 2.37E-06 | 1.64E-03 |
| Agt | 0.74 | 0.157 | 4.69 | 2.68E-06 | 1.81E-03 |
| Gm14058 | 5.40 | 1.168 | 4.62 | 3.78E-06 | 2.50E-03 |
| Baiap2l1 | 1.62 | 0.364 | 4.43 | 9.24E-06 | 5.84E-03 |
| Col18a1 | 0.94 | 0.22 | 4.30 | 1.70E-05 | 1.05E-02 |
| Plxnb3 | 0.96 | 0.226 | 4.26 | 2.09E-05 | 1.24E-02 |
| mt-Tn | 0.94 | 0.22 | 4.26 | 2.07E-05 | 1.24E-02 |
| D130058E05Rik | 3.96 | 0.957 | 4.14 | 3.46E-05 | 1.98E-02 |
| Prr5l | 1.22 | 0.296 | 4.13 | 3.66E-05 | 2.06E-02 |
| Gm25394 | 0.53 | 0.129 | 4.10 | 4.18E-05 | 2.31E-02 |
| Plek2 | 2.55 | 0.624 | 4.09 | 4.30E-05 | 2.33E-02 |
| Rnu1a1 | 0.48 | 0.117 | 4.06 | 4.97E-05 | 2.65E-02 |
| Inmt | 2.40 | 0.601 | 4.00 | 6.32E-05 | 3.25E-02 |
| Greb1 | 1.60 | 0.4 | 4.00 | 6.24E-05 | 3.25E-02 |
| Cst7 | 4.51 | 1.135 | 3.98 | 6.99E-05 | 3.54E-02 |
| Msx1 | 1.36 | 0.344 | 3.97 | 7.21E-05 | 3.59E-02 |
| Lyz2 | 1.11 | 0.282 | 3.92 | 8.71E-05 | 4.07E-02 |
| Slc4a2 | 0.93 | 0.238 | 3.92 | 8.71E-05 | 4.07E-02 |
| Gm25089 | 0.57 | 0.145 | 3.93 | 8.59E-05 | 4.07E-02 |
| Tppp3 | 0.53 | 0.134 | 3.93 | 8.43E-05 | 4.07E-02 |
| Gm22317 | 0.47 | 0.121 | 3.89 | 9.96E-05 | 4.58E-02 |
| Gm24046 | 0.85 | 0.217 | 3.89 | 1.02E-04 | 4.61E-02 |
| Gm24830 | 0.67 | 0.174 | 3.83 | 1.29E-04 | 5.66E-02 |
| Col9a3 | 1.22 | 0.32 | 3.82 | 1.36E-04 | 5.89E-02 |
| Slc16a8 | 4.81 | 1.268 | 3.79 | 1.48E-04 | 6.25E-02 |
| Tmprss5 | 1.57 | 0.413 | 3.80 | 1.46E-04 | 6.25E-02 |
| Gm25085 | 0.54 | 0.143 | 3.79 | 1.53E-04 | 6.29E-02 |
| Gm23804 | 0.38 | 0.101 | 3.78 | 1.55E-04 | 6.29E-02 |
| Syne3 | 1.16 | 0.308 | 3.77 | 1.66E-04 | 6.64E-02 |
| Neat1 | 0.67 | 0.178 | 3.76 | 1.70E-04 | 6.72E-02 |
| B230323A14Rik | 4.16 | 1.117 | 3.73 | 1.94E-04 | 7.39E-02 |
| Aspa | 1.20 | 0.322 | 3.72 | 1.96E-04 | 7.39E-02 |
| Abca4 | 1.13 | 0.303 | 3.73 | 1.94E-04 | 7.39E-02 |
| Ace | 0.87 | 0.235 | 3.72 | 1.97E-04 | 7.39E-02 |
| Gng11 | 0.99 | 0.268 | 3.72 | 2.03E-04 | 7.52E-02 |
| Prom2 | 3.46 | 0.934 | 3.71 | 2.11E-04 | 7.53E-02 |
| Nqo1 | 0.98 | 0.265 | 3.71 | 2.10E-04 | 7.53E-02 |
| Gm26444 | 0.46 | 0.124 | 3.71 | 2.07E-04 | 7.53E-02 |
| Gm23286 | 0.54 | 0.145 | 3.70 | 2.15E-04 | 7.59E-02 |
| Gm26392 | 0.46 | 0.125 | 3.69 | 2.22E-04 | 7.75E-02 |
| Cfap43 | 0.94 | 0.254 | 3.69 | 2.27E-04 | 7.82E-02 |
| Oca2 | 2.60 | 0.707 | 3.68 | 2.30E-04 | 7.84E-02 |
| Stard4 | 0.45 | 0.121 | 3.68 | 2.38E-04 | 8.02E-02 |
| Acss3 | 1.43 | 0.39 | 3.67 | 2.44E-04 | 8.09E-02 |
| Slc2a12 | 1.19 | 0.323 | 3.67 | 2.45E-04 | 8.09E-02 |
| Col4a4 | 2.14 | 0.586 | 3.66 | 2.54E-04 | 8.31E-02 |
| Il1r1 | 0.91 | 0.251 | 3.65 | 2.67E-04 | 8.64E-02 |
| Gm25790 | 0.93 | 0.255 | 3.63 | 2.81E-04 | 8.95E-02 |
| Ppfibp2 | 0.85 | 0.234 | 3.63 | 2.83E-04 | 8.95E-02 |
| Ctsd | 0.35 | 0.097 | 3.62 | 3.00E-04 | 9.40E-02 |
| Elovl7 | 1.25 | 0.345 | 3.61 | 3.09E-04 | 9.55E-02 |
| Scg5 | 0.48 | 0.134 | 3.61 | 3.12E-04 | 9.55E-02 |
| 4933406M09Rik | -18.27 | 3.158 | -5.78 | 7.30E-09 | 8.87E-06 |
| Gimap7 | -18.27 | 3.158 | -5.78 | 7.30E-09 | 8.87E-06 |
| Gm28177 | -18.27 | 3.158 | -5.78 | 7.30E-09 | 8.87E-06 |
| Gm4131 | -18.27 | 3.158 | -5.78 | 7.30E-09 | 8.87E-06 |
| RP23-56M2.5 | -18.27 | 3.158 | -5.78 | 7.30E-09 | 8.87E-06 |
| 1700067G17Rik | -18.41 | 3.157 | -5.83 | 5.56E-09 | 8.87E-06 |
| Gm19585 | -18.41 | 3.157 | -5.83 | 5.56E-09 | 8.87E-06 |
| Gpr119 | -18.41 | 3.157 | -5.83 | 5.56E-09 | 8.87E-06 |
| Mir192 | -18.41 | 3.157 | -5.83 | 5.56E-09 | 8.87E-06 |
| 4930512H18Rik | -18.62 | 3.157 | -5.90 | 3.66E-09 | 8.87E-06 |
| Traj26 | -18.62 | 3.157 | -5.90 | 3.66E-09 | 8.87E-06 |
| Slit3 | -0.46 | 0.102 | -4.46 | 8.15E-06 | 5.26E-03 |
| Kcng3 | -0.61 | 0.145 | -4.20 | 2.72E-05 | 1.59E-02 |
| Xist | -5.88 | 1.522 | -3.87 | 1.11E-04 | 4.94E-02 |
| Ccnd2 | -0.47 | 0.123 | -3.79 | 1.51E-04 | 6.26E-02 |
| Ism1 | -0.67 | 0.185 | -3.60 | 3.14E-04 | 9.55E-02 |

lfc=log_2_(fold change); lfcSE=standard error of lfc; stat=lfc/lfcSE; padj=Benjamini-Hochberg-adjusted p-value

**Supplementary Table S3** Significant GO terms output by gprofiler2

| term_id | source | term_name | padj | precision |
| --- | --- | --- | --- | --- |
| GO:0005576 | GO:CC | extracellular region | 0.003 | 0.297 |
| GO:0005615 | GO:CC | extracellular space | 0.005 | 0.234 |
| GO:0005764 | GO:CC | lysosome | 0.013 | 0.109 |
| GO:0000323 | GO:CC | lytic vacuole | 0.013 | 0.109 |
| GO:0098656 | GO:BP | anion transmembrane transport | 0.019 | 0.092 |
| GO:0061098 | GO:BP | positive regulation of protein tyrosine kinase activity | 0.019 | 0.062 |
| GO:0061097 | GO:BP | regulation of protein tyrosine kinase activity | 0.019 | 0.062 |
| GO:0045229 | GO:BP | external encapsulating structure organization | 0.019 | 0.092 |
| GO:0043068 | GO:BP | positive regulation of programmed cell death | 0.019 | 0.123 |
| GO:0043065 | GO:BP | positive regulation of apoptotic process | 0.019 | 0.123 |
| GO:0043062 | GO:BP | extracellular structure organization | 0.019 | 0.092 |
| GO:0030198 | GO:BP | extracellular matrix organization | 0.019 | 0.092 |
| GO:0006116 | GO:BP | NADH oxidation | 0.02 | 0.031 |
| GO:0030020 | GO:MF | extracellular matrix structural constituent conferring tensile strength | 0.023 | 0.052 |
| GO:1990535 | GO:BP | neuron projection maintenance | 0.031 | 0.031 |
| GO:0010942 | GO:BP | positive regulation of cell death | 0.031 | 0.123 |
| GO:0005773 | GO:CC | vacuole | 0.032 | 0.109 |
| GO:0055085 | GO:BP | transmembrane transport | 0.034 | 0.185 |
| GO:1903596 | GO:BP | regulation of gap junction assembly | 0.034 | 0.031 |
| GO:0010873 | GO:BP | positive regulation of cholesterol esterification | 0.036 | 0.031 |
| GO:0090647 | GO:BP | modulation of age-related behavioral decline | 0.036 | 0.031 |
| GO:1902950 | GO:BP | regulation of dendritic spine maintenance | 0.039 | 0.031 |
| GO:0006820 | GO:BP | anion transport | 0.039 | 0.108 |
| GO:0071914 | GO:CC | prominosome | 0.04 | 0.016 |
| GO:0120202 | GO:CC | rod photoreceptor disc membrane | 0.04 | 0.016 |
| GO:0098852 | GO:CC | lytic vacuole membrane | 0.04 | 0.063 |
| GO:0097449 | GO:CC | astrocyte projection | 0.04 | 0.031 |
| GO:0031410 | GO:CC | cytoplasmic vesicle | 0.04 | 0.188 |
| GO:0097708 | GO:CC | intracellular vesicle | 0.04 | 0.188 |
| GO:0005791 | GO:CC | rough endoplasmic reticulum | 0.04 | 0.047 |
| GO:0005783 | GO:CC | endoplasmic reticulum | 0.04 | 0.188 |
| GO:0005765 | GO:CC | lysosomal membrane | 0.04 | 0.063 |
| GO:0005604 | GO:CC | basement membrane | 0.04 | 0.047 |
| GO:0005581 | GO:CC | collagen trimer | 0.04 | 0.047 |
| GO:0012505 | GO:CC | endomembrane system | 0.04 | 0.297 |
| GO:1900272 | GO:BP | negative regulation of long-term synaptic potentiation | 0.042 | 0.031 |
| GO:0051234 | GO:BP | establishment of localization | 0.042 | 0.354 |
| GO:0045860 | GO:BP | positive regulation of protein kinase activity | 0.045 | 0.092 |
| GO:0010872 | GO:BP | regulation of cholesterol esterification | 0.045 | 0.031 |
| GO:0051179 | GO:BP | localization | 0.048 | 0.415 |
| GO:0042976 | GO:BP | activation of Janus kinase activity | 0.048 | 0.031 |
| GO:0034435 | GO:BP | cholesterol esterification | 0.048 | 0.031 |
| GO:0034433 | GO:BP | steroid esterification | 0.048 | 0.031 |
| GO:0034434 | GO:BP | sterol esterification | 0.048 | 0.031 |
| GO:0006810 | GO:BP | transport | 0.048 | 0.338 |
| GO:0001934 | GO:BP | positive regulation of protein phosphorylation | 0.048 | 0.123 |
| GO:0006801 | GO:BP | superoxide metabolic process | 0.048 | 0.046 |
| GO:0016264 | GO:BP | gap junction assembly | 0.048 | 0.031 |
| GO:0061135 | GO:MF | endopeptidase regulator activity | 0.048 | 0.069 |
| GO:0004857 | GO:MF | enzyme inhibitor activity | 0.048 | 0.086 |
| GO:0004866 | GO:MF | endopeptidase inhibitor activity | 0.048 | 0.069 |
| GO:0004925 | GO:MF | prolactin receptor activity | 0.048 | 0.017 |
| GO:0015169 | GO:MF | glycerol-3-phosphate transmembrane transporter activity | 0.048 | 0.017 |
| GO:0019807 | GO:MF | aspartoacylase activity | 0.048 | 0.017 |
| GO:0030414 | GO:MF | peptidase inhibitor activity | 0.048 | 0.069 |
| GO:0140347 | GO:MF | N-retinylidene-phosphatidylethanolamine flippase activity | 0.048 | 0.017 |
| GO:0031703 | GO:MF | type 2 angiotensin receptor binding | 0.048 | 0.017 |
| GO:0042277 | GO:MF | peptide binding | 0.048 | 0.086 |
| GO:0042802 | GO:MF | identical protein binding | 0.048 | 0.224 |
| GO:0140323 | GO:MF | solute:anion antiporter activity | 0.048 | 0.034 |
| GO:0102707 | GO:MF | S-adenosyl-L-methionine:beta-alanine N-methyltransferase activity | 0.048 | 0.017 |
| GO:0098615 | GO:MF | dimethyl selenide methyltransferase activity | 0.048 | 0.017 |
| GO:0030748 | GO:MF | amine N-methyltransferase activity | 0.048 | 0.017 |
| GO:0004790 | GO:MF | thioether S-methyltransferase activity | 0.048 | 0.017 |
| GO:0097386 | GO:CC | glial cell projection | 0.049 | 0.031 |

padj=FDR-adjusted pval; precision=the proportion of genes in the input list that are annotated to the function; BP=biological process; CC=cellular component; MF=molecular function

**Supplementary Table S4** Differentially expressed genes and genes linked to differentially methylated CpG sites.

| Category | Genes |
| --- | --- |
| Upregulated genes (84) | 2010110K18Rik, 2210010C04Rik, Abca4, Ace, Acss3, Agr2, Agt, App, Aspa, B230323A14Rik, Baiap2l1, Bpifa6, Cfap43, Col18a1, Col4a4, Col9a3, Cst7  Ctsd, D130058E05Rik, D930032P07Rik, Elovl7, Gfap, Gm10375, Gm11313, Gm11771, Gm14058, Gm15169, Gm15658,Gm17171, Gm22317, Gm23275, Gm23286, Gm23804, Gm24046, Gm24135, Gm24305, Gm24368, Gm24515, Gm24830, Gm25085, Gm25089  Gm25394, Gm25790, Gm25813, Gm26147, Gm26232, Gm26392  Gm26444, Gm8215, Gng11, Greb1, H2-M10.6, Il1r1, Inmt, Lyz2, Msx1, mt-Tn, Neat1, Nqo1, Oca2, Olfr314, Plek2, Plxnb3, Ppfibp2, Prlr, Prnp, Prom2, Prr5l, Rnu1a1, RP23-402K6.2, RP24-329K22.1, Scg5, Slc16a8, Slc2a12, Slc37a2, Slc4a2, Ssxb2, Stard4, Syne3, Tdpoz4, Tff3, Tmprss5, Tppp3, Xlr3e-ps |
| Downregulated genes (16) | 1700067G17Rik, 4930512H18Rik, 4933406M09Rik, Ccnd2, Gimap7, Gm19585, Gm28177, Gm4131, Gpr119, Ism1, Kcng3, Mir192, RP23-56M2.5, Slit3, Traj26 |
| Hypermethylated genes (71) | 7530416G11Rik, Adcy8, Arfgef1, Arl6ip6, Atp2b2, C1qtnf5, Ccdc167, Ccdc96, Cela2a, Cep192, Col9a2, Crhr2, Cryab, Crym, Cyp2c67, Defb33, Dlgap4, Ect2l, Epc2, Fam50b, Fam72a, Fstl4, Fzd8, Ggnbp1, Gjd4, Gm14092, Gpr146, Hexim1, Hsd17b8, Hspa12b, Hspb1, Il1rap, Inafm1, Irx2, Isx, Lbx1, Lpp, Mab21l1, Magea14, Mamdc2, Map1a, Mif, Mtpn, Myo18b, Nnat, Nphp3, Nrxn2, Or2w4, Or7g33, Plagl1, Prkacb, Psmd7, Rab11fip5, Rai1, Ranbp9, Rassf5, Rbm20, Sarm1, Scn5a, Shisa2, Slc22a1, Sox13, Spock1, Tdrp, Tex43, Timeless, Traf1, Trim36, Vmn2r82, Zc3h15, Zfp366 |
| Hypomethylated genes (111) | Abcb7, Ablim3, Acsl4, Arfip1, Arx, Arxes1, Atf3, Atp7a, Bcap29, Bclaf3, C1ql3, Cacng3, Camk2n1, Ccdc85c, Cdkn2a, Commd8, Cspg4, D430019H16Rik, Dgat1, Diaph2, Dnajb8, Dock11, Drd2, Duox1, Eef2, Erich2, F11r, Fgf16, Frrs1l, Ftsj1, Gpr25, Hoxc9, Hspg2, Ihh, Ints12, Iqcj, Iqsec2, Kat6a, Kcna6, Lin7b, Ltf, Lyn, Magee1, Mamld1, Man1a2, Map3k9, Map7, Mecp2, Med14, Mkx, Mmp16, Mpp1, Mroh1, Mtmr3, Nhs, Nhsl2, Nlgn2, Olig1, Or4x6, Oxt, Pax3, Pcdh7, Pdk3, Pgr, Phlpp1, Pld1, Pmaip1, Prr12, Rai2, Rasgrf2, Rbmxl2, Rhov, Rilpl1, Rragc, Scx, Sh3kbp1, Shmt2, Shroom2, Slc22a3, Slc25a14, Slc27a2, Slc30a3, Slc6a8, Smc1a, Sox14, Sox3, Sp6, Spata20, Spef1, Speg, Sstr4, Stk26, Tbl1x, Tbx1, Tmem200c, Tmem47, Trim72, Trpc5, Ttn, Ube2q1, Vma21, Vps54, Xk, Zfp185, Zfp280c, Zfp382, Zfp449, Zfp534, Zfp992, Zkscan17, Zmym3 |

Genes starting with 'Gm-' or ending with 'Rik' are annotated genes that do not have a canonical name (yet).

**Supplementary Table S5** Genes that are commonly present in each latent variable component pair

| Component | Genes |
| --- | --- |
| RNA component 1 | diablo_r1.csv in Supplementary Data |
| DNA methylation component 1 | Diablo_d1_gene.csv |
| Genes common between RNA component 1 and DNA methylation component 1 | Entrez ID 14950 = H13  Official Full Name = histocompatibility 13  Predicted to enable aspartic endopeptidase activity, intramembrane cleaving; protein homodimerization activity; and ubiquitin protein ligase binding activity |
| RNA component 2 | diablo_r2.csv |
| DNA methylation component 2 | Diablo_d2_gene.csv |
| Genes common between RNA component 2 and DNAm component 2 | Entrez ID 12653 = Chgb  Official Full Name = chromogranin B  Predicted to localize in extracellular region. Predicted to be active in extracellular space and secretory granule.  Entrez ID 332934 = Zmynd12  Official Full Name = zinc finger, MYND domain containing 12  Orthologous to human ZMYND12 (zinc finger MYND-type containing 12)  Entrez ID 338521 = Fa2  Official Full Name=fatty acid 2-hydroxylase  Enables fatty acid alpha-hydroxylase activity. Involved in galactosylceramide biosynthetic process; glucosylceramide biosynthetic process; and plasma membrane raft organization. Acts upstream of or within several processes, including lipid modification; myelin maintenance; and sebaceous gland cell differentiation.  Entrez ID 433931 = Pigg  Official Full Name=phosphatidylinositol glycan anchor biosynthesis, class G  Predicted to enable CP2 mannose-ethanolamine phosphotransferase activity. Predicted to be involved in GPI anchor biosynthetic process. Predicted to be in endoplasmic reticulum.  Entrez ID 629059 = Fam124a  Official Full Name=family with sequence similarity 124, member A  Orthologous to human FAM124A (family with sequence similarity 124 member A) |

**Supplementary Figure S1**


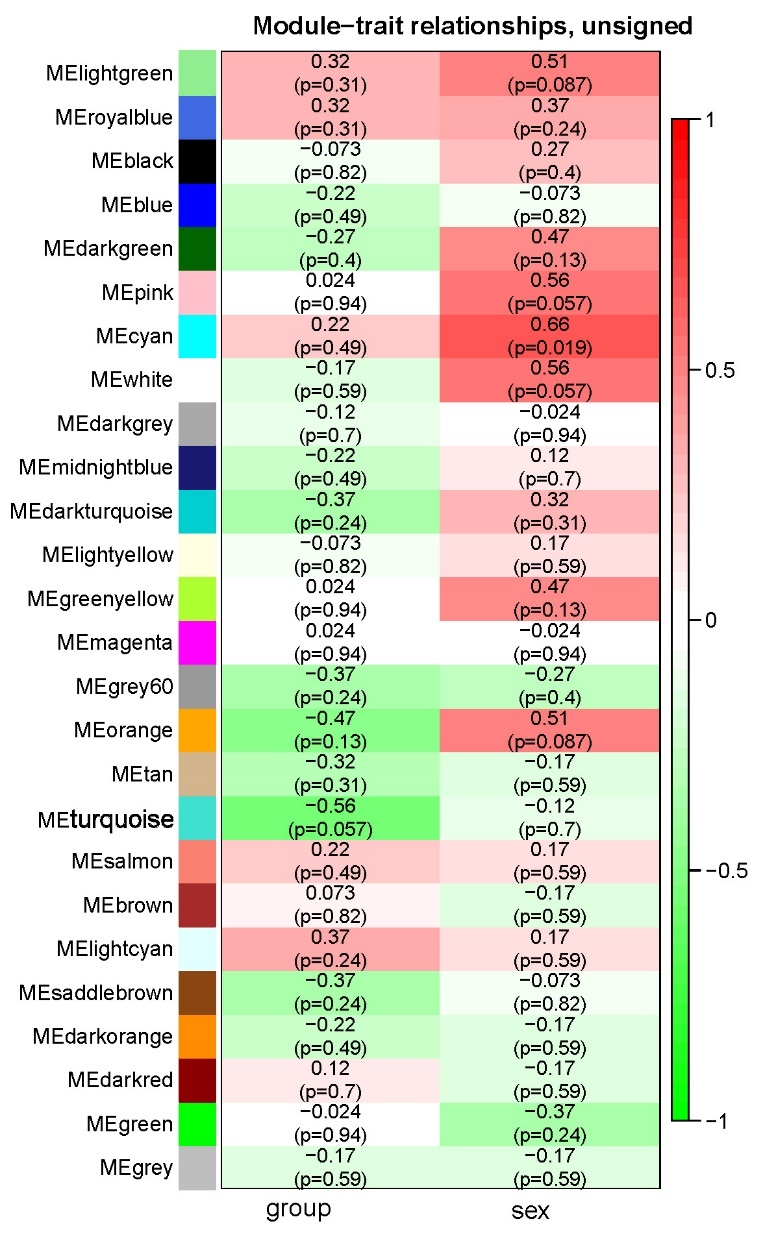


Module-trait associations using an unsigned gene network. Rows and columns represent eigengenes and traits, respectively. The group column is for Tg vs. Wt and sex is for female vs. male. The number in each cell represents Spearman’s correlation coefficient, along with its pval (p). Cells are colored according to their correlation coefficients.

**Supplementary Figure S2**


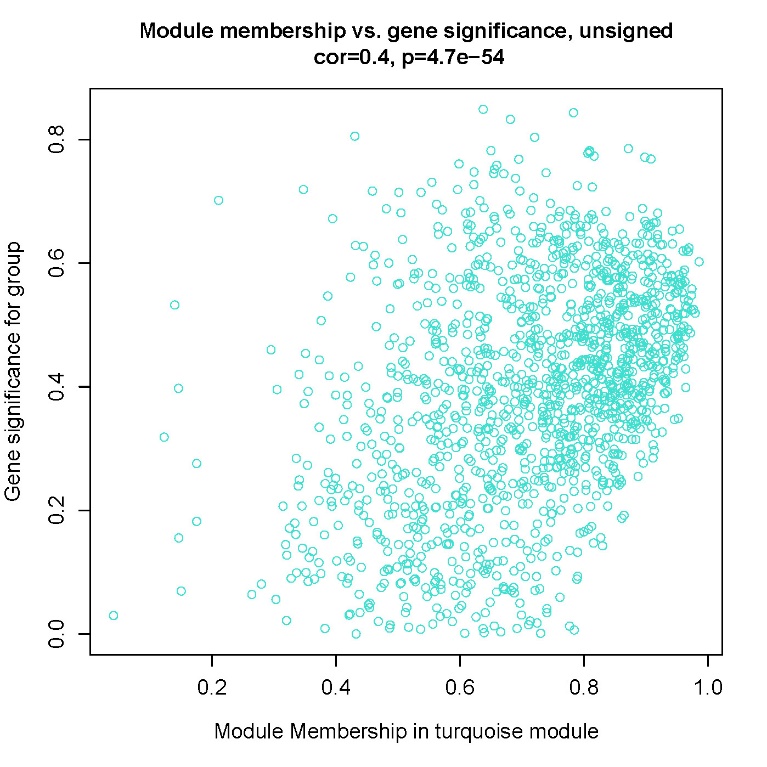


A scatterplot of gene significance vs module membership in the turquoise module. The two are significantly correlated, with Pearson’s correlation coefficient (cor)=0.4, and pval (p) close to 0.

**Supplementary Figure S3**


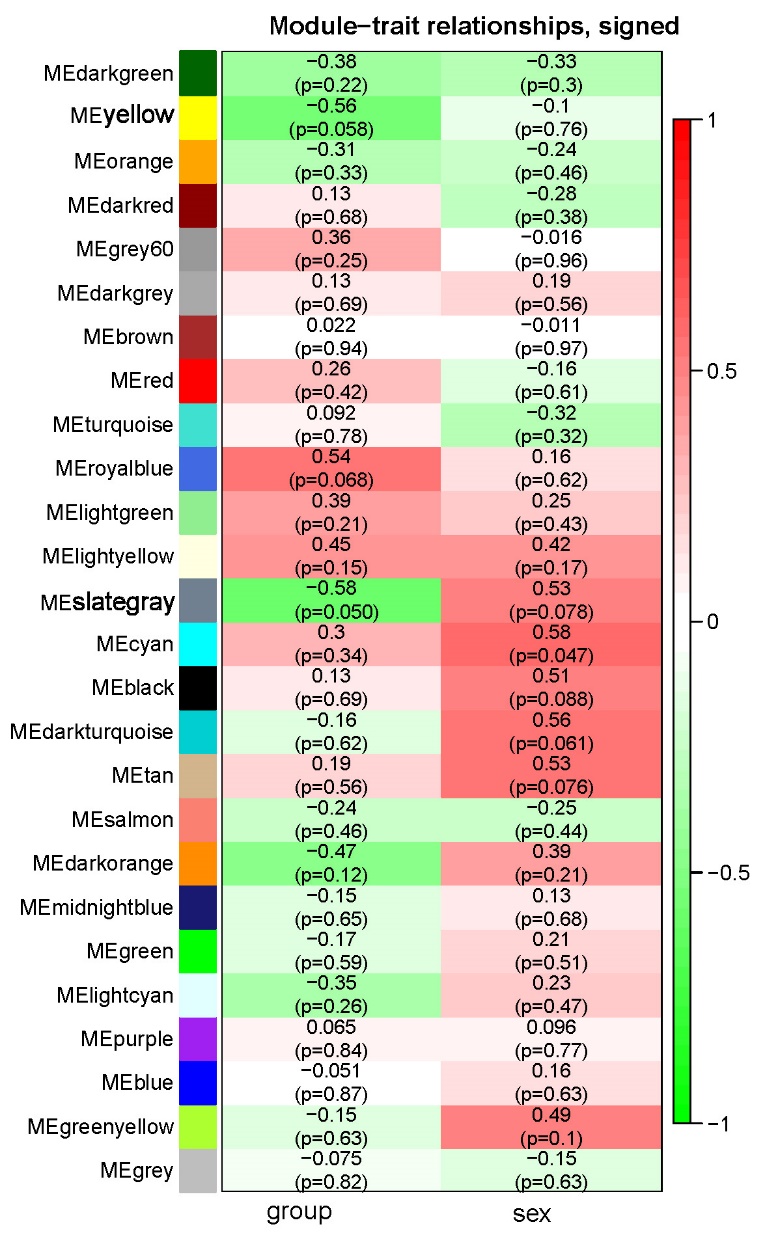


Module-trait associations using a signed gene network. Rows and columns represent eigengenes and traits, respectively. The group column is for Tg vs. Wt and sex is for female vs. male. The number in each cell represents Spearman’s correlation coefficient, along with its pval (p). Cells are colored according to their correlation coefficients.

**Supplementary Figure S4**


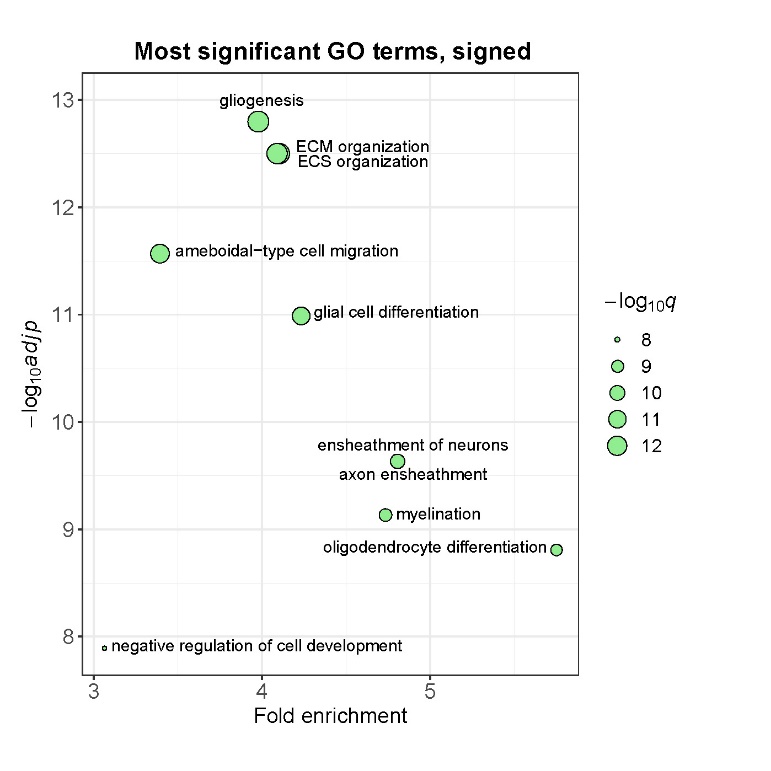


A bubble plot of top 10 GO terms enriched among the genes in the yellow module. Fold enrichment is the ratio of the proportion of input genes annotated in each GO term to the proportion of all genes annotated in the same term; padj=Benjamini-Hochberg-adjusted pval; q=FDR-adjusted pval. All terms in the plot belong to the biological process category.

**Supplementary Figure S5**


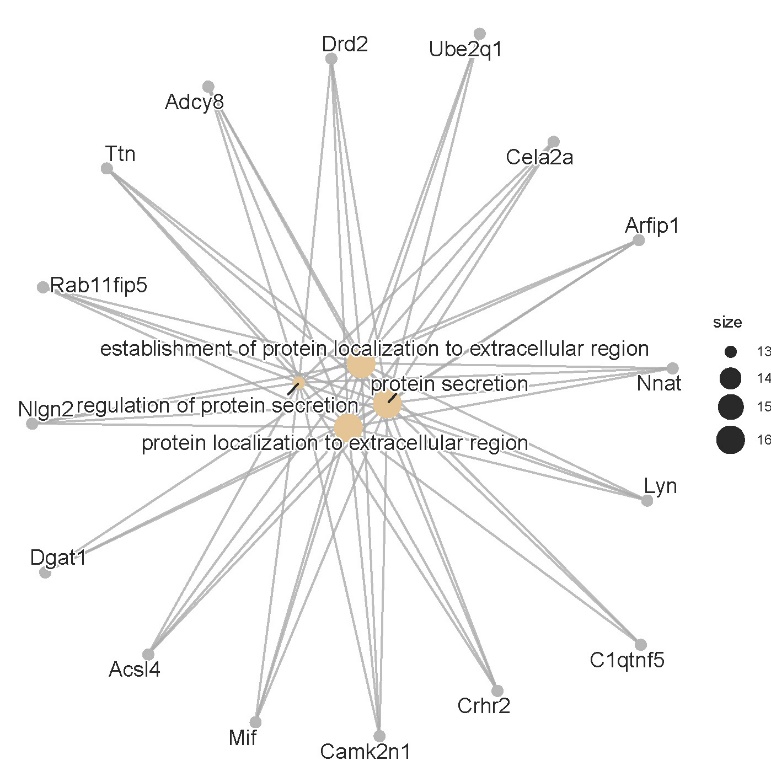


A “cnetplot” that displays the top four GO terms (Table 2) and related genes in the input gene list. It was generated using the cnetplot function available in the R enrichplot package. “size” refers to the number of genes assigned to each GO category

# B. Supplementary Methods

Note that all raw datasets and how they were processed are available in GEO repository with accession numbers GSE223417 for RNA-seq and GSE223348 for BS-seq.

B.1. RNA-seq

RNA preparation was done in one batch, but library preparation and sequencing were done in two batches (batches 1 and 2 in B.1.5 below) because each library kit can accommodate a maximum of 8 samples. Each sample is tagged with a unique sample-specific barcode; so, samples were pooled together for cost-effective sequencing. The average number of total reads was 33.2 million per sample, which included all non-unique alignments. Following preprocessing and quality control, the average number of unambiguous, unique reads was 18 million per sample (B.1.2). The entire output from DESeq2 contains data from 30,372 genes (2.4 Mb in size), but most of them are not significant at all, with their p-val = 1. Only 100 of them are significant as differentially expressed genes between the control and Tg mice. In case, however, we uploaded a manageable Excel file containing 1,685 genes, including the top 100, and relevant statistical values (Deseq_out_1685.csv in Supplementary Data).

B.1.1. Downloading transcript count data from Ion Torrent Server

B.1.2. Transformation of Count data to an edgeR object using the DGEList function in the edgeR package

ID group lib.size* norm.factors sample_names sex

12 WT 16936130 1 12 F

32 TG 19496827 1 32 F

41 WT 18824542 1 41 M

67 TG 17781219 1 67 M

68 TG 19229417 1 68 M

69 TG 20995113 1 69 M

79 WT 21042580 1 79 F

80 WT 16251483 1 80 F

91 TG 18762170 1 91 F

92 TG 15803401 1 92 F

93 TG 17931268 1 93 F

95 WT 17994452 1 95 M

*The total number of unambiguous, unique reads

B.1.3. The edgeR object was analyzed by the R WGCNA package as directed by the package

# WGCNA recommends using unfiltered and unnormalized data

B.1.4. Filtering and normalization of data using *calcNormFactors* and *cpm* functions in edgeR

B.1.5. Setting up Design matrix using the model.matrix function in R DESeq2 package

model.matrix(~0+sex+batch+group)

sexF sexM batch2 groupWT

1 1 0 0 1

2 1 0 0 0

3 0 1 0 1

4 0 1 0 0

5 0 1 1 0

6 0 1 1 0

7 1 0 1 1

8 1 0 1 1

9 1 0 0 0

10 1 0 0 0

11 1 0 0 0

12 0 1 0 1

B.1.6. Selection of differentially expressed genes (DEG) using *DESeqDataSetFromMatrix, DESeq,* and *results* functions as directed by DESeq2 (Deseq_out_1685.csv)

---

out of 30377 with nonzero total read count

adjusted p-value < 0.1

LFC > 0 (up) : 84, 0.28%

LFC < 0 (down) : 16, 0.053%

outliers [1] : 5, 0.016%

low counts [2] : 0, 0%

---

B.1.7. 100 DEGs (deseq2_Deg.csv)

B.2. DNA methylomics using the R methylKit package

The raw DNA methylation dataset containing data for all 12 samples is 894 MB in size. Following the methylKit pipeline, the raw data were re-organized, using the unite function, to cover only the methylation sites that are covered in all samples. The resulting united dataset is 132 MB in size. After applying the 10% cutoff for absolute value of methylation percentage change and qvalue cutoff of 0.1, we have 443 differentially methylated sites (Diff_10per.csv). Positive meth.diff values indicate hypermethylation in Tg mice and negative meth.diff hypomethylation.

B.2.1. Reading in processed Bam files provided by ZymoResearch using *methRead*

obj.brn.11 = methRead(report.files, sample.id = list('12', '32','41', '48','67','68', '69', '79', '80', '91', '92', '93'),

assembly = "mm10", context = "CpG", treatment = c(0, 1, 0, 1, 1, 1, 1, 0, 0, 1, 1, 1))

# treatment = 0 (Wt) or 1 (Tg)

# obj.brn.11 is ~ 894 MB in size

B.2.2. Combining methyl data using the *unite* function

meth.brn.11 = unite(obj.brn.11, destrand = FALSE)

meth.brn.11, 131 MB in size, contains information for 981,122 DNA methylation sites for 11 samples.

B.2.3. Selection of Methy CpG sites where absolute methylation difference ≥ 10%, qvalue ≤ 0.1, and covariate adjustment for sex specified as ('F','F','M','M','M','M','M','F','F','F','F','F') using *calculateDiffMeth* and *getMethylDiff* functions (‘F’ = female and ‘M’=male)

# in Diff.10p.csv, positive meth.diff indicates hypermethylation in Tg mice and negative meth.diff indicates hypomethylation

B.2.4. Annotation of each methyl sites *using biomaRt and org.Mm.eg.db* packages

B.2.5. GO over-representation using *enrichGO* in clusterProfiler and *cnetplot* in enrichplot packages.

B6. Multi-block Discriminant Analysis DIABLO (Data Integration Analysis for Biomarker discovery using a Latent cOmponents)

RNA and DNA methylation datasets from 11 samples were analyzed using the *block.splsda* function in the R mixOmics package. The parameters include two input datasets (x1=RNA data; x2=DNA methylation data) and the response variable (y = Wt or Tg). The goal was to select 500 features for the first two components that discriminate between the two groups of samples. The two components were selected using selectVar function (diablo_r1.csv, diablo_r2.csv, diablo_d1_raw.csv, diablo_d2_raw.csv in Supplementary Data) and gene names (diablo_d1_gene.csv, diablo_d2_gene.csv) of the selected DNA methylation sites were retrieved using *biomaRt and org.Mm.eg.db* packages.

# C. Supplementary Data

Supplementary_Data.zip contains the following files:

dms_443-gene_182.cvs

Diff_10per.csv

diablo_r1.csv

diablo_r2.csv

diablo_d1_raw.csv

diablo_d1_gene.csv

diablo_d2_raw.csv

diablo_d2_gene.csv

Deseq_out_1685.csv

ego_10p.csv

ego_diablo_r2.csv
